# Antioxidant and anti-inflammatory effects of selenium in oral buccal mucosa and small intestinal mucosa during intestinal ischemia-reperfusion injury

**DOI:** 10.1186/s12950-014-0036-1

**Published:** 2014-10-30

**Authors:** Yongsoo Kim, Dong Chil Kim, Eui-Sic Cho, Seung-O Ko, Woon Yong Kwon, Gil Joon Suh, Hyo-Keun Shin

**Affiliations:** Department of Oral & Maxillofaical Surgery, School of Dentistry and Institute of Oral Bioscience, Research Institute of Chonbuk National University Hospital, Chonbuk National University, 664-14, Duckjindong, Chonju, 561-756 Chonbuk Korea; Department of Emergency Medicine, Seoul National University College of Medicine, 101 Daehak-Ro Jongno-Gu, Seoul, 110-744 Korea

**Keywords:** Ischemia reperfusion injury, Selenium, Lipid peroxidation, NF kappaB, Buccal mucosa, Small intestine

## Abstract

**Background:**

The aim of this study were to investigate whether selenium treatment attenuates lipid peroxidation and downregulates the NF-κB pathway in small intestinal mucosa and to examine whether the effect of selenium is also observed in oral buccal mucosa, during small intestinal IR injury.

**Materials and methods:**

Eighteen rats were assigned into three groups: sham, IR, and IR + selenium. Saline or selenium was administered through a tail vein. 24 hours later, the superior mesenteric artery was exposed and clamped in the IR and IR + selenium groups. After ischemic and reperfusion period, animals were sacrificed and oral buccal mucosa and small intestinal mucosa were harvested.

**Results:**

Glutathione peroxidase activity and cytoplasmic IκB-α expression was higher in the IR + selenium group than that in the IR group. A malondialdehyde level, cytoplasmic phosphorylated inhibitor κB-α, nuclear NF-κB p65 expressions, and NF-κB p65 DNA-binding activity were lower in the IR + selenium group than those in the IR group.

**Conclusion:**

A selenium treatment may cause increased GPx activity, attenuated lipid peroxidation, and downregulated the NF-κB pathway during small intestinal IR injury. Furthermore, these therapeutic benefits of selenium can be observed in oral buccal mucosa as well as small intestinal mucosa.

## Introduction

When the blood supply diminishes in metabolically active tissues, the tissue ischemia results in the so-called ischemic injury. Subsequently, when the blood flow restores, the reperfusion injury also occurs. This reperfusion injury can damage the affected tissues more seriously than the ischemic injury [1]. The development of reperfusion injury is mediated by reactive oxygen species (ROS) formation, lipid peroxidation, and inflammatory responses [2].

Among the internal organs, the small intestine is the most vulnerable organ to ischemia-reperfusion (IR) injury [3]. IR injury in the small intestine breaks mucosal barrier, which results in occurrence of systemic inflammatory response syndrome (SIRS). Then, SIRS can induce secondary injuries to the distant organs leading to multiple organ failure (MOF) or even to death [4]. The mechanisms of the initiation and progression of SIRS during small intestinal IR injury are ROS formation, ROS-dependent inhibitor κB-α (IκB-α) phosphorylation in cytoplasm, and subsequent nuclear factor κB (NF-κB) activation in nucleus. Recent *in vitro* studies have shown that ROS enhances cytoplasmic signaling pathways leading to NF-κB nuclear translocation [5-7]. Therefore, the antioxidant therapy can be considered as a therapeutic strategy to attenuate inflammatory responses induced by IR injury. Previous studies also reported that selenium suppressed NF-κB activation [8,9].

Selenium is an essential element having antioxidant and immunomodulation function. Selenium is used to synthesize the amino acid, called selenocysteine, which is vital to the function of selenoprotein. At least, 25 selenoproteins are found in the human body and those are classified by the function into antioxidant enzymes (glutathione peroxidase, GPx), antioxidant proteins (selenoprotein P and W), other metabolic enzymes (thioredoxin reductase and iodine deiodinase) [10]. Previous preclinical studies have reported that selenium suppresses ROS formation by increasing GPx activity [11,12]. This antioxidant effect could be responsible for the beneficial effect of selenium such as inhibition of carcinogenesis, myocardial protection in myocardial infarction, neuronal protection in focal neurological injury [13,14]. Furthermore, some clinical studies showed that lower selenium level was related with the increased mortality and the administration of selenium (sodium selenite, Na_2_O_3_Se) has improved the survival [15,16]. However, there have been no *in vivo* studies which investigate therapeutic effects of selenium on small intestinal IR injury.

The most accurate method to determine therapeutic effects of selenium on small intestinal IR injury is to examine the small intestine itself, which is clinically very difficult. Oral buccal mucosa has the similar state of differentiation to that of the small intestinal villi and shows the decreased microvascular blood flow in distributive shock [17-19]. Furthermore, oral buccal mucosa can be easily and noninvasively accessible in clinical setting. If oral buccal and small intestinal mucosal damages during intestinal IR injury are similar, the oral buccal mucosa can be considered as a marker for the evaluation of intestinal mucosal injury. In fact, our previous study was conducted to determine the usefulness of oral buccal mucosa in the evaluation of the intestinal IRI by estimating the activation level of the NF-кB pathway of the buccal mucosa, and the result suggested a possibility of the buccal mucosa as an indicator for the assessment of intestinal ischemia-reperfusion injury [20]. Therefore, we hypothesized that selenium pretreatment would enhance GPx activity, attenuate lipid peroxidation, and downregulate the NF-кB pathway by suppression of ROS formation and subsequent ROS-dependent IκB-α phosphorylation during small intestinal IR injury, and that antioxidant and anti-inflammatory effects of selenium would be observed in oral buccal mucosa as well as small intestinal mucosa.

The purpose of this study were to examine whether selenium pretreatment is associated with the enhancement of GPx activity, the suppression of ROS formation and subsequent ROS-dependent IκB-α phosphorylation, the attenuation of lipid peroxidation, and the downregulation of the NF-кB pathway during small intestinal IR and to investigate if antioxidant and anti-inflammatory effects of selenium are observed in oral buccal mucosa as well as small intestinal mucosa during small intestinal IR injury.

## Materials and methods

### Experiment animals

Experiments were performed on male Sprague–Dawley rats (body weight, 300 – 350 g; Orient Bio Incorporated, Seongnam, Republic of Korea). Animals were maintained on a laboratory chow (Lab Diet) and water *ad libitum* and housed in a specific pathogen-free room at constant temperature (20 - 22°C) with 10 and 14 hrs of light and dark exposure, respectively. Animals underwent an acclimatization period of 14 days before being used in experiments. All of the experiments conducted were approved by the Institutional Animal Care and Use Committee of our institute.

### Experimental procedure

Subjects were divided into the 3 groups (n = 6 in each group): the sham group, animals of which underwent a sham operation; the IR group, ischemic-reperfusion (IR) injury alone; the IR + selenium group, IR injury plus selenium infusion. Experimental procedures to induce IR injury were performed as previously described [20]. Briefly, rats were anesthetized with 100% oxygen and 3% Isoflurane (Forane solution; Choongwae Pahrmacy, Seoul, Republic of Korea) in the rodent circuit controller. Appropriate level of anesthesia was obtained by intramuscular injection of 15 mg/kg of Zoletil (zolazepam/tiletamine; Virbac Labaratories, Carros cedex, France) in the femur of rats. A midline incision was made in abdominal wall and the superior mesenteric artery (SMA) was isolated without any damage to the peritoneal organs and blood vessels. In the IR and IR + selenium group, the isolated SMA was clamped with a bulldog clamp. Adequate blockage of SMA was confirmed by the color of the intestine turned pale (please refer to the figure in our previous report) [20]. To minimize water and heat loss, abdominal muscle and skin layers were then closed. After the 30 minutes of ischemic period, laparotomy was carried out again to restore SMA blood flow. The bulldog clamp was removed and reperfusion of the SMA was verified by the scarlet color of the small intestine. Abdominal muscle and skin layers were closed again. Then the animals were returned to their cages and allowed food and water *ad libitum.* All animals received 50 mg/kg of subcutaneous normal saline fluid after the procedures and no antibiotics were administered.

In the sham group, the clamping and declamping of the SMA were not performed. The IR + selenium group was administered selenium (60 μg/kg) and the sham and IR group were administered saline vehicle through a tail vein 24 hours before the procedures. The rationale to administer selenium at 24 hours before intestinal IR injury was based on the our previous study which showed therapeutic benefits of selenium at 24 hours after administration in paraquat intoxicated rats [12].

After the 30 minutes of ischemic period and the 90 minutes of reperfusion period, animals were sacrificed and oral buccal mucosa and small intestinal mucosa were harvested. Oral buccal mucosa of rats was obtained from both cheeks. Separated oral buccal mucosa and small intestinal mucosa were washed with phosphate buffered solution (PBS) for three times. After drying, those were frozen and stored in liquid nitrogen tank at −70°C until required. Blood samples obtained by cardiac puncture were centrifuged at 2,000 g for 15 min at 4°C, and separated sera were stored at −80°C for subsequent assays.

### Measurements of GSH and GSSG levels, GPx activity, and MDA level

Reduced glutathione (GSH) and oxidized glutathione (GSSG) levels in oral buccal mucosa and small intestinal mucosa were measured using a glutathione assay kit (Cayman Chem. Ann Arbor, MI, USA) [21]. Glutathione peroxidase (GPx) activity was measured using a glutathione peroxidase assay kit (Cayman Chem). Malondialdehyde (MDA) level was fluorometrically measured as 2-thiobarbituric acid-reactive substance by the Ohkawa method [22,23].

### Nuclear and cytoplasmic extracts

The extraction of nuclear and cytoplasmic proteins from oral buccal mucosa and small intestinal mucosa were performed using a NEPER nuclear and cytoplasmic extraction reagents kit (Pierce Chemical, Rockford, IL) [24]. Protein contents in the supernatant of the lysed mucosa were determined using a BCA protein assay reagent kit (Pierce Chemical).

### Western blot analysis

To determine the expressions of cytoplasmic phosphorylated IκB-α (p-IκB-α) and IκB-α and that of NF-κB p65, we performed Western blotting as previously described [24]. In brief, nuclear and cytoplasmic extracts (10 μg per lane) were run on 8% and 12% sodium dodecyl sulphate-polyacrylamide gels, respectively, and then transferred to polyvinylidene difluoride membranes (Schleicher & Schuell, Dassel, Germany). The following primary antibodies (Cell Signaling, Beverly, CA) were used for immunoblotting: rabbit antirat-p-IκB-α (1:250), IκB-α (1:500), and NF-κB p65 (1:500) antibodies. As secondary antibodies, antirabbit immunoglobulin G (Stressgen, Victoria, BC, Canada) coupled with peroxidase and diluted 1:2000 in tris-buffered saline-Tween were used. Protein bands were detected by an enhanced chemiluminescence (ECL) system (Amersham International, Buckinghamshire, UK). Optical densities were quantified by a computer-assisted densitometric analysis of the exposed films (Lap Work Software; Seoulin Bioscience, Seoul, Republic of Korea). All blots were normalized against β-actin to control for protein loading. For β-actin measurements, the mentioned Western blot method was applied using a specific mouse monoclonal anti-β-actin antibody (Sigma-Aldrich, St.Louis, MO, USA).

### NF-κB P65 DNA-binding activity

NF-κB p65 DNA-binding activity was determined using the TransAM method, and a NF-κB p65 transcription factor assay kit (Active Motif, Carlsbad, CA) was used to detect and quantify NF-κB transcription factor activation in oral buccal mucosa and small intestinal mucosa [25].

### Enzyme-linked immunosorbent assay

Serum tumor necrosis factor-α (TNF-α) level was measured using Duoset enzyme-linked immunosorbent assay kit (R&D System, Minneapolis, MN). Enzyme-linked immunosorbent assay plates were measured using a Versa Max microplate reader (Molecular Devices Corp, Sunnyvale, CA) at 450 nm and concentrations of the respective proteins in serum were calculated according to calibration curves.

### Statistical analysis

Data were analyzed by the Kruskal-Wallis with Mann–Whitney *U post hoc* test and Bonferroni correction. *P*-values of <0.017 were considered statistically significant and the significance levels quoted are two-sided. Statistical analysis was conducted using SPSS version 12.0 for Windows (SPSS, Chicago, IL).

## Results

### GSH and GSSG levels and GPx activity

There were no significant differences in GSH and GSSG levels in oral buccal mucosa and small intestinal mucosa between the IR and IR + selenium groups. In both the oral buccal mucosa and small intestinal mucosa, however, GPx activity was higher in the IR + selenium group than in the IR group (*p* = 0.004 and 0.002, respectively) (Table 1).

**Table 1:** **Laboratory results**

**N = 6/group. Median (range)**						
	**Small intestine**			**Oral buccal mucosa**		
	**Sham**	**IR**	**IR + selenium**	**Sham**	**IR**	**IR + selenium**
GSH level (pmol/mg tissue)	460 (404–580)	400 (385–455)	401 (339–427)	509 (444–555)	468 (449–540)	448 (429–478)
GSSG level (pmol/mg tissue)	397 (322–457)	382 (348–419)	447^✝^(418–467)	387 (337–438)	354 (321–399)	392 (360–416)
GPx activity (mU/mg tissue)	3.06 (2.63-3.12)	2.72 (2.34-2.94)	3.47^*✝^(3.20-3.82)	3.26 (2.90-3.34)	2.60^*^(2.30-2.77)	2.90^✝^(2.76-3.16)
MDA level (pmol/mg tissue)	118 (110–134)	1113^*^(849–1258)	395^*✝^(178–471)	41 (21–58)	119^*^(106–134)	94^*✝^(48–108)
p-IκB-α expression (OD)	1.00 (0.53-1.28)	1.87^*^(1.49-2.50)	1.06^✝^(0.60-1.29)	0.23 (0.07-0.35)	1.45^*^(1.04-2.42)	0.52^✝^(0.31-0.83)
IκB-α expression (OD)	1.00 (0.88-1.28)	0.22^*^(0.12-0.44)	0.92^✝^(0.68-1.56)	0.48 (0.37-0.65)	0.20^*^(0.20-0.36)	0.48^✝^(0.32-0.58)
NF-κB p65 expression (OD)	1.00 (0.78-1.70)	17.40^*^(9.93-17.69)	3.77^*✝^(3.63-4.62)	0.33 (0.08-0.59)	8.44^*^(7.15-9.93)	2.32^*✝^(1.73-3.16)
NF-κB p65 DNA-binding (OD)	1.00 (0.90-1.23)	2.65^*^(1.80-2.93)	1.62^*✝^(1.20-2.03)	0.47 (0.47-0.50)	0.59^*^(0.54-0.67)	0.51^✝^(0.47-0.54)
	Sham		IR		IR + selenium	
Serum TNF-α level (pg/mL)	32.35 (31.08-34.90)		226.60 (183.46-330.04)		155.33 (129.01-203.32)	

*GSH*, Reduced glutathione; *GSSG*, Oxidized glutathione; *GPx*, Glutathione peroxidase; *MDA*, Malondialdehyde; *p-IκB-α*, Phosphorylated inhibitor kappa B-α; *NF-κB*, Nuclear factor kappa B; *IR*, Small intestinal ischemia-reperfusion injury; *TNF-α*, Tumor necrosis factor α.

^*^ *p* <0.017 vs. the sham group. ^✝^ *p* <0.017 vs. the ischemic-reperfusion injury (IR) group.

### MDA Level

In the IR + selenium group, MDA level was lower than that in the IR group in both the oral buccal mucosa and small intestinal mucosa (*p* = 0.004 and 0.002, respectively) (Table 1).

### Cytoplasmic p-IκB-α and IκB-α expressions

**Figure 1:**
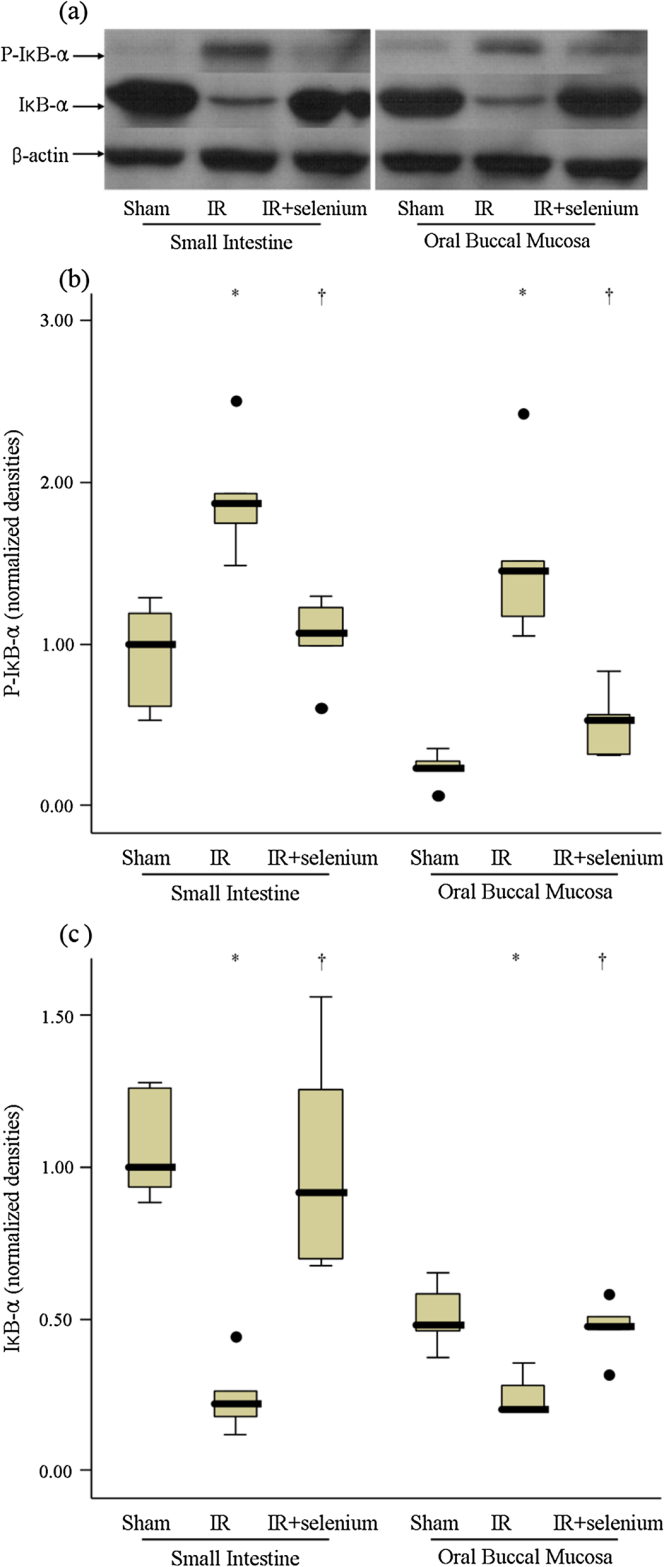
**Cytoplasmic phosphorylated inhibitor κB-α (p-IκB-α) and IκB-α expressions in oral buccal mucosa and small intestinal mucosa.** Blots **(a)** are representative of the results of 6 rats per group. Normalized densities of p-IκB-α **(b)** and IκB-α **(c)** expressions. Data are median (quatile, range). ^*^ *p* <0.017 vs. the sham group. ^✝^ *p* <0.017 vs. the ischemic-reperfusion injury (IR) group.

In the IR + selenium group, cytoplasmic p-IκB-α expression in oral buccal mucosa and small intestinal mucosa was lower than that in the IR group (*p* = 0.008 and 0.008, respectively) (Figure 1). In contrast, cytoplasmic IκB-α expression was higher in the IR + selenium group than that in the IR group in both the oral buccal mucosa and small intestinal mucosa (*p* = 0.016 and 0.008, respectively) (Figure 1).

### Nuclear NF-кB p65 expression

**Figure 2:**
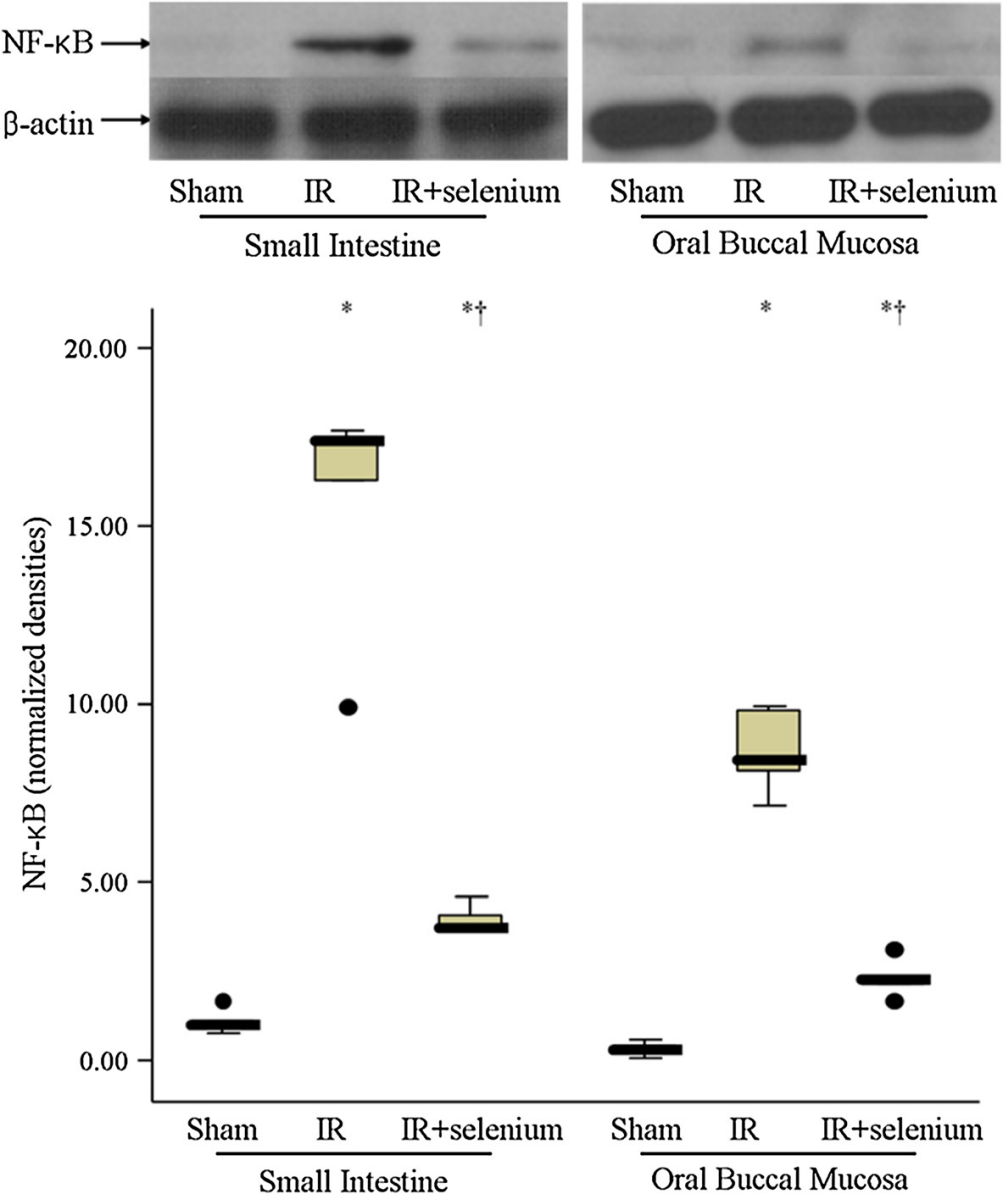
**Nuclear NF-κB p65 expression in oral buccal mucosa and small intestinal mucosa.** Blots are representative of the results of 6 rats per group. Data are median (quatile, range). ^*^ *p* <0.017 vs. the sham group. ^✝^ *p* <0.017 vs. the ischemic-reperfusion injury (IR) group.

In the IR + selenium group, nuclear NF-кB p65 expression was lower than that in the IR group in both the oral buccal mucosa and small intestinal mucosa (*p* = 0.008 and 0.008, respectively) (Figure 2).

### NF-кB p65 DNA-binding activity

**Figure 3:**
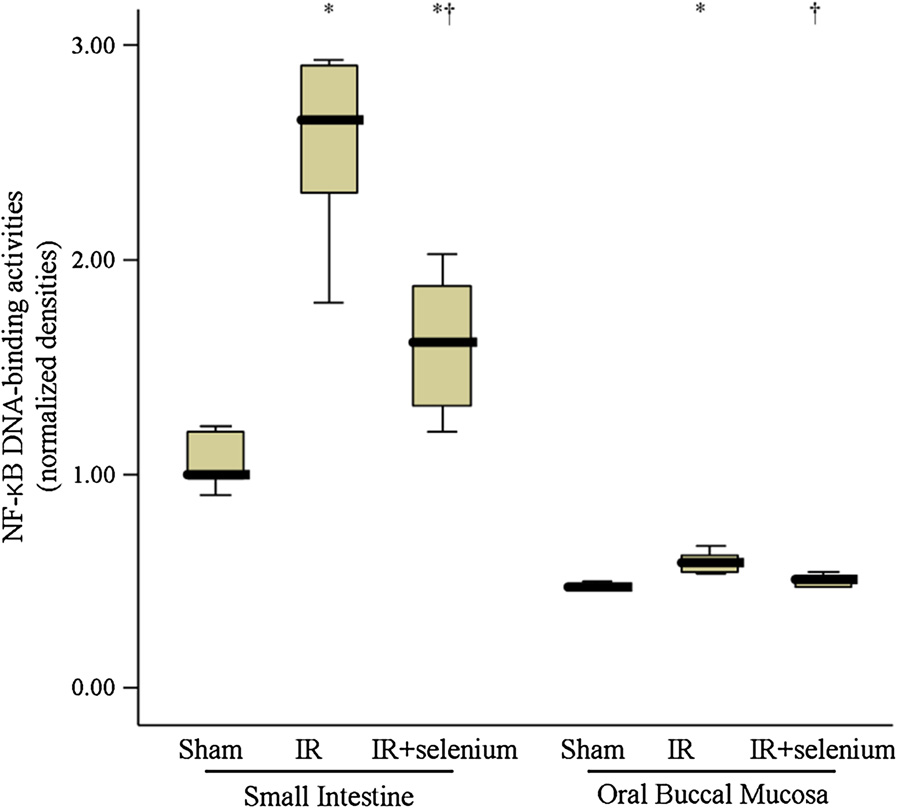
**NF-κB p65 DNA-binding activity in oral buccal mucosa and small intestinal mucosa.** Data are median (quatile, range). ^*^ *p* <0.017 vs. the sham group. ^✝^ *p* <0.017 vs. the ischemic-reperfusion injury (IR) group.

NF-кB p65 DNA-binding activity in oral buccal mucosa and small intestinal mucosa was also lower in the IR + selenium group than that in the IR group (*p* = 0.004 and 0.009, respectively) (Figure 3).

### Serum TNF-α level

**Figure 4:**
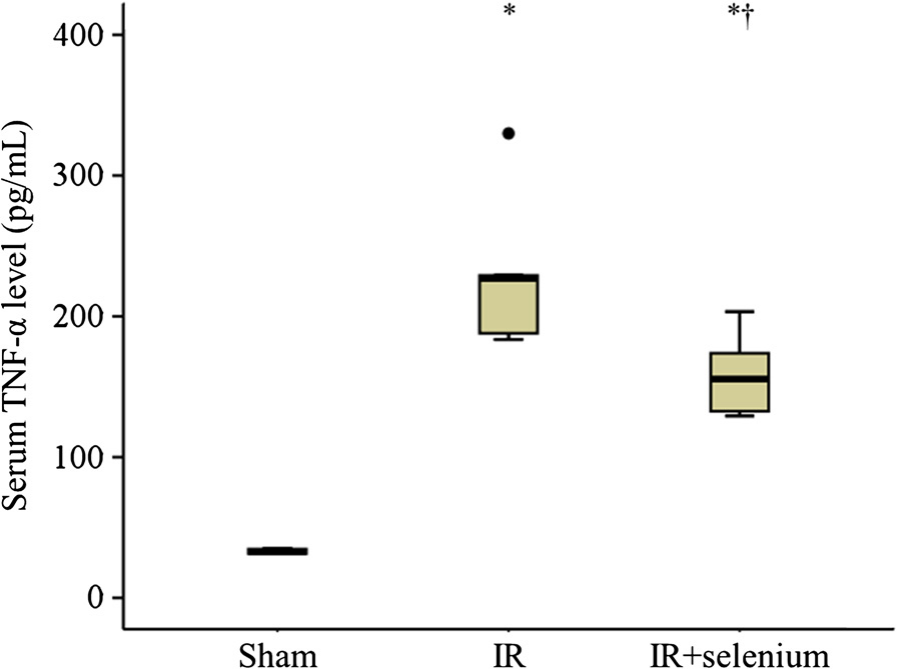
**Serum TNF-α level.** Data are median (quatile, range). ^*^ *p* <0.017 vs. the sham group. ^✝^ *p* <0.017 vs. the ischemic-reperfusion injury (IR) group.

Serum TNF-α level in the IR + selenium group was significantly lower than that in the IR group (*p* = 0.009) (Figure 4).

## Discussion

In the present study, we found that selenium pretreatment increased GPx activity, attenuated lipid peroxidation, downregulated the NF-κB pathway during small intestinal IR injury. Previous *in vitro* experiments have reported that oxidative stresses impair GPx to an inactive form and GPx activity has a primary role in the defense against oxidative stresses [26,27]. Our data also showed that small intestinal IR injury suppressed GPx activity and the increase of selenium-dependent GPx activity by selenium treatment contributed to attenuation of lipid peroxidation and downregulation of the NF-κB pathway. Although the mechanisms of downregulation of the NF-κB pathway by selenium were not fully determined in this study, we suggest that increased GPx activity eliminates ROS and suppresses subsequent ROS-dependent NF-κB activation. Since we did not use living tissues but frozen tissues, we were not able to measure ROS directly. We indirectly measured MDA level as a substitute for ROS. MDA has been known as a marker for lipid peroxidation by ROS. The decreased MDA levels in selenium treated rats in this study might be caused by the suppressed ROS formation, which support our suggestion.

In the present study, selenium treatment did not alter GSH or GSSG levels, which failed to show significant differences between the IR and selenium treated IR groups. Glutathione reductase reduces GSSG to GSH, and GPx oxidized GSH to GSSG. No change of GSH or GSSG levels in our study suggests that alternative mechanisms may be related to maintain GSH level regardless of increased GPx activity. Other previous data have shown that an adaptive response to ROS results in producing an increase in GSH level and the mechanism is associated with recovery of glutathione reductase activity or increased expression of γ-glutamylcysteine synthase [28,29]. Further studies which measure these enzymes will be needed to confirm our data.

Small intestinal IR injury occurs in the various medical conditions including necrotizing enterocolitis, midgut volvulus, intussusception, mesenteric ischemic disease, small bowel transplantation, aortic aneurysm surgery, cardiopulmonary bypass, hemodynamic shock, and sepsis. In the present study, therapeutic benefits of selenium were consistent in both the oral buccal mucosa and small intestinal mucosa. These results indicate that as a substitute for the intestinal mucosa, oral buccal mucosa can be used to detect the development of oxidative or inflammatory damages and to evaluate the beneficial effects of therapeutic agents in critically ill patients with small intestinal IR injury. In previous experimental data, we also found that NF-κB pathway was upregulated in both the oral buccal mucosa and the small intestinal mucosa during small intestinal IR injury [20]. These data indicate that antioxidant effects of selenium as well as oxidative stresses can be determined in oral buccal mucosa during small intestinal IR injury. However, the MDA level and NF-κB p65 DNA-binding activity of small intestinal mucosa were higher than those of oral buccal mucosa in the IR and selenium treated IR groups. These data suggest that the development of oral buccal mucosal injury is due to systemic inflammatory responses rather than direct IR injury.

Recent meta-analysis data suggest that high dose of selenium (>500 μg/day) may improve the outcomes of critically ill patients, particularly dose at high risk of death [30-33]. Clinical trials of selenium supplementation have used a wide range of doses. In several studies, selenium was administered as an initial loading dose of 1000 – 2000 μg and thereafter a continuous infusion of 1000 – 1600 μg/day [16,34]. However, in two recent large randomized controlled studies (RCT), 500–800 μg/day of selenium were administered [35,36]. We infused a single dose of selenium (60 μg/kg). This dose of selenium in rats corresponds well to the doses used in the recent RCTs [35,36]. Furthermore, the human dose can be translated to experimental dose in rat by the body surface area normalization method [37,38]. The dose of selenium in rat would then range from 48 to 77 μg/kg. Based on this calculation, we selected a 60 μg/kg as a clinically relevant dose. Clinically, it is so difficult to determine the adequate timing of selenium administration after small intestinal IR injury for the maximal antioxidant effect of selenium. The present study shows that the GPx activities can be measured in oral buccal mucosa, directly. Before its clinical use, further studies using more clinically relevant model should be performed.

In the present study, the amount of tissues of oral buccal mucosa was very small, and we failed to analyze tissue directly. An additional set of experiment for morphological tissue analysis is needed to confirm our data.

## Conclusion

Selenium enhanced glutathione peroxidase activity, attenuated lipid peroxidation, and downregulated the NF-κB pathway during small intestinal ischemic-reperfusion injury. Furthermore, these therapeutic benefits of selenium seem to be observed in oral buccal mucosa as well as small intestinal mucosa.

## Abbreviations

GPx: Glutathione peroxidase
GSH: Reduced glutathione
GSSG: Oxidized glutathione
IκB-α: Inhibitor κB-α
IR: Ischemia-reperfusion
MDA: Malondialdehyde
MOF: Multiple organ failure
NF-κB: Nuclear factor κB
PBS: Phosphate buffered solution
p-IκB-α: Phosphorylated IκB-α
ROS: Reactive oxygen species
SIRS: Systemic inflammatory response syndrome
SMA: Superior mesenteric artery
